# Medication‐related incidents at 19 hospitals: A retrospective register study using incident reports

**DOI:** 10.1002/nop2.534

**Published:** 2020-05-31

**Authors:** Maria Cottell, Inger Wätterbjörk, Maria Hälleberg Nyman

**Affiliations:** ^1^ Department of Patient Safety Örebro University Hospital Örebro Sweden; ^2^ School of Health Sciences Faculty of Medicine and Health Örebro University Örebro Sweden

**Keywords:** hospital, incident reporting, medication errors, nursing, patient safety, register study

## Abstract

**Aim:**

To examine (a) when medication incidents occur and which type is most frequent; (b) consequences for patients; (c) incident reporters’ perceptions of causes; and (d) professional categories reporting the incidents.

**Design:**

A descriptive multicentre register study.

**Methods:**

This study included 775 medication incident reports from 19 Swedish hospitals during 2016–2017. From the 775 reports, 128 were chosen to establish the third aim. Incidents were classified and analysed statistically. Perceived causes of incidents were analysed using content analysis.

**Results:**

Incidents occurred as often in prescribing as in administering. Wrong dose was the most common error, followed by missed dose and lack of prescription. Most incidents did not harm the patients. Errors in administering reached the patients more often than errors in prescribing. The most frequently perceived causes were shortcomings in knowledge, skills and abilities, followed by workload. Most medication incidents were reported by nurses.

## 1 INTRODUCTION

Medication administration is a multidisciplinary responsibility and a process in many steps that requires several types of professional knowledge (Ben Natan, Sharon, Mahajna, & Mahajna, 2017). It is one of the most frequently performed nursing tasks, accounting for about 16% to 40% of all nursing work (Potter et al., 2005; Westbrook, Duffield, Li, & Creswick, 2011). Medication administration in clinical practice is becoming more complex as a result of diversification of medication routes and medical devices and increasing severity of patients’ conditions. Therefore, a large percentage of adverse events are related to medication errors (Ben Natan et al., 2017).

Studies have shown high frequencies of errors in medication (Barker, Flynn, Pepper, Bates, & Mikeal, 2002; Cronenwett, Bootman, Wolcott, & Aspden, 2007; Phillips et al., 2001). In 2007, the U.S. Institute of Medicine estimated that 1.5 million people are subjected to medication‐related errors every year, leading to an additional cost of 3.5 billion dollars and to 98,000 deaths in the USA (Cronenwett et al., 2007). In Sweden, approximately a third of avoidable adverse events are due to errors in medication (Soop, Fryksmark, Koster, & Haglund, 2009). According to the Institute of Medicine, medication‐related errors are a significant cause of morbidity and mortality (U.S. Institute of Medicine, 1999).

## 2 BACKGROUND

Medication error is defined by the National Coordinating Council for Medication Error Reporting and Prevention (NCC MERP) as any preventable event that may cause or lead to inappropriate medication use or patient harm, while the medication is under the control of the healthcare professional, patient or consumer (The National Coordinating Council for Medication Error Reporting and Prevention, 2014).

Errors can occur during any phase of the drug delivery process, from prescription to drug administration and at any place where medications are administered (Fortescue et al., 2003). The term medication incident (MI) is often used. This refers to both medication errors reaching the patient and near misses prevented before an error has happened. Reporting of MIs has been recognized as a useful tool in understanding why errors occur and planning opportunities for error prevention (Costello, Torowicz, & Yeh, 2007; Joolaee, Hajibabaee, Peyrovi, Haghani, & Bahrani, 2011). Several countries have national incident reporting systems (IRSs) (Cheung, van den Bemt, Bouvy, Wensing, & De Smet, 2011; Holmstrom et al., 2012; Yürür & Valdez, 2018). Healthcare staff in Sweden are governed by the Patient Safety Act (Ministry of Health & Social Affairs, 2010) and are obliged to report risks of adverse events and near misses.

Earlier observational studies have examined the incidence of MIs in terms of administration errors made by nurses, for example, wrong time, omission or failure to follow guidelines (Blignaut, Coetzee, Klopper, & Ellis, 2017; Kim & Bates, 2013) and show the complexity in working with medication administration (Bucknall et al., 2019). Another study on severe medical incidents caused by nurses and reported to the Swedish National Board for Health and Welfare identified six main categories of errors: (a) wrong dose, (b) wrong drug, (c) dose(s) missed, (d) unauthorized or unordered drug, (e) wrong route and (f) drug administered despite documented allergy (Bergqvist, 2012). The most common severe medication errors were “wrong dose” (41%), “wrong patient” (13%) and “omission of drug” (12%) (Björkstén, Bergqvist, Andersén‐Karlsson, Benson, & Ulfvarson, 2016).

Data from IRSs have been used to analyse how MIs are detected in different phases of medication handling (Harkanen, Turunen, Saano, & Vehvilainen‐Julkunen, 2015; Jylhä, Bates, & Saranto, 2016). However, not much is known about the prevalence, distribution, seriousness, consequences and causes of less severe MIs taking place on an everyday basis in Swedish hospitals and reported in the different IRSs. These incidents have the potential to cause harm of varying seriousness to patients. It is vital to understand MIs to plan improvement strategies. If the systems are to effectively drive improvements that reduce harm, organizations must not only collect events, but also categorize the frequency, types and causes associated with the incidents (Ioannidis & Lau, 2001; Leape, Berwick, & Bates, 2002; Mekhjian, Bentley, Ahmad, & Marsh, 2004). The aims of this study were to examine (a) the phase of medication handling where incidents in hospitals occur and the most frequent errors, using a web‐based IRS; (b) consequences for patients; (c) incident reporters’ perceptions of causes; and (d) professional categories reporting the incidents.

## 3 METHODS

### 3.1 Design

The present study was a descriptive, multicentre register study on MI reports, including quantitative data analyses. We used MI reports from five county councils (CC) in Sweden to assess the phase of medication handling where incidents in hospitals occur and the most frequent errors, using a web‐based IRS; the consequences for patients; the reporters’ perceptions of causes; and professional categories reporting the incidents. The study adhered to the STrengthening the Reporting of OBservational studies in Epidemiology (STROBE) guideline for observational studies (Appendix S1).

### 3.2 Sample

MI reports from the 19 hospitals in five CCs in Sweden were included. Of the 19 hospitals, two were university hospitals, 11 county hospitals and 6 local hospitals serving in total approximately 1 400 000 people.

All the hospitals in these CCs used the same web‐based IRS and the reports included where and when the incident took place, a written account of what took place, the damage/injuries that happened or could have happened and, in free text, an opinion about the cause of the incident.

The time frame was 30 June–31 December 2016. One of the CCs did not have the same version of the IRS until 2017, and therefore, data were obtained for the period 1 January to 30 June 2017. After written consent from the managers at the different CCs, data were extracted from the IRS in each CC by a local administrator. The total number of incident reports (IRs) received was 1,397. Incidents pertaining to storage, supply, lack of medicine and control of narcotic drugs were excluded from the study. The first author (MC) read all IRs and excluded the ones not meeting the inclusion criteria, leaving 775 MI reports to be included in the study. The data held no information regarding the identity of patients or reporters.

MI reports from only one CC held enough data to enable investigation of reporters’ perceptions of causes (aim 3). Therefore, a second selection from the original 775 was made including these 159 MI reports. After excluding the ones that contained insufficient data regarding the cause or stating: “cause not known,” 128 MI reports remained.

### 3.3 Ethical considerations

An application was sent to the Uppsala/Örebro Region Ethics Committee. Since the data in the study did not contain any information regarding identity of patients or reporters, it was considered that no formal ethical scrutiny was required. The Board issued an advisory remark stating no ethical concerns in carrying out the study (Reference no: 2017/162).

### 3.4 Data analysis

The 775 MI reports were read in full and analysed. To determine in what phase of medication handling incidents occurred and to identify the most frequent errors, we used the categorization already made in the IRS. The MI reports were sorted into four main phases of medication handling: prescription, medication list, preparation and administration. To determine the most frequent errors, the reporters’ free‐text descriptions were used and categories were developed from these. Kruskal–Wallis H test was used to discover differences in proportions between the five CCs, and chi‐square test was used to conclude statistical significance between categorical variables. A *p* value of less than .05 was considered statistically significant. Analyses were performed using IBM SPSS statistics version 24 for Windows (IBM Corp., Armonk, NY, USA).

We used the NCC MERP Medication Error Index to determine the consequences for patients. This taxonomy includes nine categories (categories A–I) that reflect whether an actual error occurred (actual error, categories B–I) or not (potential error, category A), whether the error reached the patient (categories C–I) or not (categories A–B) and whether the error resulted in harm to the patient (harmful error, categories E–I) or not (no harmful error, categories A–D) (The National Coordinating Council for Medication Error Reporting and Prevention, 2014).

To summarize the reporters’ free‐text descriptions of the perceived causes of the incidents, quantitative content analysis was used (Krippendorff, 2004). The 128 selected MI reports’ free‐text descriptions were analysed and categorized and sorted under predetermined headings and subheadings in the framework for analysing risk and safety in clinical medicine (Vincent, Taylor‐Adams, & Stanhope, 1998). All reports were first read in whole and scrutinized by the first author (MC) and sorted into the framework. The co‐authors (IW & MHN) viewed the sorting, and consistency was achieved. This increases the credibility of the findings (Polit & Beck, 2020). As the reports were analysed, four new subheadings were developed: inexplicit protocol, deviating from protocol, inadequate control and communication with patient. To ensure transferability (Polit & Beck, 2020), examples of the text in the reports and the coding are presented in Tables 3 and 4. To ensure trustworthiness, all authors critically reviewed the data analysis process. To enhance confirmability (Polit & Beck, 2020), the authors’ pre‐understanding was taken into consideration and the authors strove to be open towards the text.

## 4 RESULTS

### 4.1 Errors in different phases of medication handling

The analysis of the 775 MI reports showed no significant differences between the CCs in the distribution of phases in medication handling where the incidents took place (*p* = .406). Most incidents happened during administration (44.3%, *N* = 343), followed by prescribing (40.6%, *N* = 315). There were no significant differences between these two phases (*p = *.241). Incidents in managing the medication list amounted to 10.7% (*N* = 83) and in preparation 3.6% (*N* = 28). In the administration phase, wrong dose was the most common error (*N* = 82). The second most common error was missed single dose (*N* = 61). This applied to all the five CCs. Also in prescribing, wrong dose was the most common error in all CCs but one (*N* = 89). Missing or incomplete prescription was the second largest error reported in total (Table 1).

**TABLE 1:** Distribution of medication‐related incidents in five Swedish county councils in different phases of medication handling (*N* = 775)

	CC 1 *N* = 182	CC 2 *N* = 159	CC 3 *N* = 119	CC 4 *N* = 130	CC 5 *N* = 185	Total *N*	Mean
Administration *N* (%)	83 (45.6)	71 (44.6)	47 (39.5)	67 (51.5)	75 (40.5)	343	44.3%
Wrong dose	19	16	17	14	16	82	
Missed single dose	15	14	8	12	12	61	
Mix‐up of drugs	8	14	5	7	11	45	
Wrong time	8	5	4	7	10	34	
Wrong patient	7	4	1	2	3	17	
Wrong drug	2	2	7	9	3	23	
Missed signing	5	3	1	9	9	27	
Wrong route	3	1	1	0	0	5	
Handling drug	5	4	2	3	2	16	
Missed dose (>1)	2	0	0	3	4	9	
Overdose	4	1	0	0	2	7	
Wrong rate	4	2	1	1	2	10	
Patient hypersensitive to drug	1	1	0	0	1	3	
Not checking ID	0	1	0	0	0	1	
Expired drug given	0	3	0	0	0	3	
Prescribing *N* (%)	64 (35)	64 (40)	49 (41)	54 (41.5)	84 (45.4)	315	40.6%
Wrong dose	10	18	12	19	30	89	
Prescription is lacking	13	10	11	17	16	67	
Prescription incomplete	7	6	14	5	4	36	
Prescription inexplicit	13	8	1	1	9	32	
Wrong time	4	4	2	6	6	22	
Wrong drug	4	7	0	3	5	19	
Drug prescribed to allergic patient	5	2	2	2	1	13	
Missed release	2	3	1	0	4	10	
Prescription lost between units	3	2	2	1	1	9	
Wrong patient	1	2	1	0	3	7	
Patient not informed	0	0	3	0	2	5	
Wrong route	1	1	0	0	2	4	
Prescribing interacting drugs	1	1	0	0	1	3	
Medication list *N* (%)	24 (13)	11 (7)	19 (16)	7 (5.3)	22 (11)	83	10.7%
New drug/change in dose/released drug not documented (single drug)	4	1	10	3	0	18	
Information lost in patient transfer	3	3	3	2	2	13	
List not activated	0	4	1	0	5	10	
Drug missing on list	3	1	3	0	2	9	
List not updated (more than one drug)	3	0	2	1	2	8	
Other	11	2	0	1	11	25	
Preparation *N* (%)	6 (3)	9 (5)	4 (33)	2 (1.5)	7 (4)	28	3.6%
Wrong concentration	4	0	1	1	0	6	
Infusion not labelled	1	3	0	0	5	9	
Mix‐up risk	0	4	0	0	0	4	
Wrong diluent	1	0	1	0	0	2	
Wrong drug	0	1	0	0	0	1	
Concentrate not added	0	1	0	1	1	3	
Wrong ID labelling	0	0	1	0	0	1	
Transfusion not documented	4	0	1	0	0	5	

Abbreviation: CC, county council

### 4.2 Consequences to patients

In 740 of the 775 IRs, it was possible to determine the consequence to the patient using the NCC MERP Medication Error Index (Table 2). Categories C (error reached the patient but no harm; 47.1%, *N* = 349) and B (error occurred but did not reach the patient; 38.6%, *N* = 286) were the most frequent. In the administration phase, most incidents pertained to category C (67.5%, *N* = 223) and in the prescribing phase to category B (58.8%, *N* = 177). In all, 96.3% (*N* = 713) of the incidents rendered no harm to patients (categories A–D) and 3.7% (*N* = 27) rendered harm of varying seriousness (categories E, F; Table 2). Table 3 shows examples of the reporters’ descriptions of consequences.

**TABLE 2:** Consequences of errors, NCC MERP Medication Error Index (*N* = 740), for patients in different phases of medication handling

	Administration	Prescribing	Medication list	Preparation	Total *N* (%)
No error, *N* (%)					
A ‐ Circumstances or events that have the capacity to cause error	1 (0.3)	0	0	1 (3.7)	2
Error, no harm, *N* (%)					
B ‐ Error occurred, but the medication did not reach the patient	47 (14.2)	177 (58.8)	55 (67)	7 (25.9)	286 (38.6)
C ‐ Error occurred that reached the patient, but did not cause the patient harm	223 (67.5)	84 (27.9)	18 (21.9)	18 (66.6)	349 (47.1)
D ‐ Error occurred that resulted in the need for increased patient monitoring, but not patient harm	45 (13.6)	28 (9.3)	8 (9.7)	2 (7.4)	83 (11.2)
Error, harm, *N* (%)					
E ‐ Error occurred that resulted in the need for treatment or intervention and caused temporary patient harm	13 (3.9)	7 (2.3)	0	0	20 (2.7)
F ‐ Error occurred that resulted in prolonged hospitalization and caused temporary patient harm	2 (6)	5 (1.6)	1 (1.2)	0	8 (1.1)
G ‐ Error occurred that resulted in permanent patient harm	0	0	0	0	0
H ‐ Error occurred that resulted in a near death event (e.g. anaphylaxis, cardiac arrest)	0	0	0	0	0
Error, death, *N* (%)					
I ‐ Error occurred that resulted in patient death	0	0	0	0	0
Total	330	301	82	27	740

**TABLE 3:** Examples of the reporters’ descriptions of consequences and how they were assessed

“Patient safety is at risk if we don't have the right protocol for the infusion pumps to administer Ketanest! We don't use it on a regular basis and therefore we are not accustomed to using it!”	A
“The Patient is on Warfarin. This was not prescribed either today or yesterday.” ”Infusion of antibiotics not signed.” “Patient due for operation has been prescribed different preoperative drugs in the web‐system and the anaesthesia chart.”	B
“The patient was supposed to get Oxycodone 5 mg but got Oxycodone Depot 5 mg. The boxes look the same and so do the pills.” “When I started my evening shift, I discovered that the antibiotics supposed to be administered at 10 or 11 a.m. wasn't signed. I called the day‐shift nurse and the dose had been missed.” “Wrong prescription of Methotrexate. Was prescribed as 15 mg/day but should have been 15 mg/week. The patient realized the error after having swallowed the pills.”	C
”Patient with COPD. Desaturates to SPo2 74% and several controls of SPo2 and arterial blood gases are performed during the day. When I start my evening shift I discover that the patient is attached to air, not oxygen. Patient retains normal SPo2 once the oxygen is attached.” “Hectic morning. I take Insulin from the patient's medication box. There were 3 types of insulin in the box and after administering I discover that I took the short‐acting insulin when it should have been the long‐term acting. Controls of blood sugar level are performed several times.”	D
“After the enteral nutrition was turned off the iv Insulin was not turned off. The patient suffered a drop in blood glucose level and had to have an infusion with Glucose 300 mg/mL iv.” “Patient has a mechanical heart valve and is treated with Warfarin. Blood samples today show too high INR‐ 5.6. The patient is currently on Trimethoprim, which could explain the elevated INR as it interacts with Warfarin. Thus, the patient was prescribed this drug without having been scheduled for extra control of the INR level. Warfarin was released and after a couple of days the therapeutic level of Warfarin then got too low making it necessary to prescribe Fragmin until therapeutic levels can be restored. This mistake has caused risks and inconvenience to the patient.”	E
“The patient has been using Omeprazole for some time. After release from the ward, Omeprazole has somehow disappeared from the medication list. Possibly he hasn't been getting this medication for several weeks. A few days ago the patient started complaining about difficult chest pain and contacted the ward. We recommended he go to the ER if the pain didn't wear off which he did. It turned out the pain was related to gastric problems due to not getting the Omeprazole. The patient has many serious conditions and is in poor shape and the visit to the ER was very strenuous for him. It was also unnecessary and due to a mistake on our side.”	F

Note

A: Circumstances or events that have the capacity to cause error. B: Error occurred, but the medication did not reach the patient. C: Error occurred that reached the patient, but did not cause the patient harm. D: Error occurred that resulted in the need for increased patient monitoring, but not patient harm. E: Error occurred that resulted in the need for treatment or intervention and caused temporary patient harm. F: Error occurred that resulted in prolonged hospitalization and caused temporary patient harm

### 4.3 Reporters’ perceptions of causes of medication incidents

The single largest perceived cause was shortcomings in knowledge, skills and abilities (*N* = 47; Table 4). Inexplicit protocols, deviating from protocol and inadequate verifying together represented the single largest group (*N* = 56). The second largest perceived cause was workload (*N* = 30). In 19 cases, the error was ascribed to insufficient communication between colleagues and co‐workers. Of the 128 reports, 125 (97.6%) were made by Registered Nurses (RNs) and three (2.4%) by physicians. In physician‐made mistakes, the causes were mainly ascribed to lack of knowledge, skills and abilities. The mistakes made by nurses were more often ascribed to high workload and working environment.

**TABLE 4:** Examples of how reporters’ descriptions of causes of medication incidents were sorted into the framework for analysing risk and safety in clinical medicine

Examples of reporters’ narratives	Sorted under Heading/Subheading	*N*
	**Patient characteristics**	**1**
“Long and extensive medication list due to patient's complex condition”	Condition (complexity and seriousness)	
	**Individual (staff) factors**	**47**
“Inexperienced physician not accustomed to the EMR and EHR” “The nurse in charge of printing out medication records before the system upgrade has not been responsible for this and not been given sufficient information about the procedure” “The prescribing physician is an intern, relatively inexperienced and probably thought about the child's age (12 years) instead of weight when prescribing the drug”	Knowledge, skills, abilities	
	**Team factors**	**26**
“Miss in communication between student and supervising nurse” ”Not enough support in decision making”	Supervision and seeking help	2
“Misunderstanding between senior physicians on standby from different units” “Communication lacking between physicians and in reporting the patient to the ward” “Miss in communication. The staff taking over the patient did not know an intravenous infusion was ongoing”	Communication with colleagues (verbal and/or written)	19
“Inexplicit/inadequate information from physician to the patient that the strength and dose of the injection had been changed” “Patient has not had or not understood information to stop treatment with EOX 21 days after Oxaliplatin treatment	Communication with patient†	5
	**Task factors**	**56**
“Different systems for documenting (paper and electronic)	Task design and clarity of structure	2
“Unclear routines regarding where to prescribe pre‐medication, since the old chart for anaesthesia still is in use” “Unclear routines when changing an as‐needed medication to a standing medication.”	Inexplicit protocol†	19
“This is the result of prescribing on paper and not in the electronic prescribing system.” “Different prescriptions in several places: anaesthesia chart, electronic system”	Deviating from protocol†	10
“Not double checking which type of intravenous Kabiven is supposed to be administered.” “Patients identity cannot have been checked before starting the infusion, since the ID number was wrong”	Inadequate verifying†	25
	**Work environment**	**37**
“Lack of staff and lack of time are contributing factors to this incident” “The head nurse was inexperienced”	Staffing levels and skills mix	4
“I realize that I missed giving the patient his medicine. The morning was very hectic and I forgot” “The incident may have happened due to stress that led to insufficient control before administering the drug” “Stress‐related causes. Did not note that the IV line wasn't completely attached”	Workload	30
“Fault in infusion device. Device returned to supplier”	Design, availability and maintenance of equipment	3

^†^ Subheadings were developed by the first author (MC).

## 5 DISCUSSION

### 5.1 Errors in different phases of medication handling

Consistent in all CCs, most reported incidents pertained to the administration and prescribing phases. Earlier studies also suggest prescribing and administration to be associated with the greatest number of medication errors (Buckley, Erstad, Kopp, Theodorou, & Priestley, 2007; Kopp, Erstad, Allen, Theodorou, & Priestley, 2006). The administration stage of the medication process is especially vulnerable to error because errors are least likely to be caught before reaching the patient. Medication administration is not simply the giving of drugs and nor is it clearly defined; medication administration entails a complex mixture of varied and often competing demands that temporally structure the nurses’ entire workday (Jennings, Sandelowski, & Mark, 2011). Also, the nurse is often interrupted while administering the medication (Bucknall et al., 2019). Interruption is known to increase the risk for MIs, and it is essential to provide a work environment that reduces the interruptions (Hayes, Jackson, Davidson, & Power, 2015). The results of this study show that MIs occur to the same extent in the prescribing and administering phases. In the administration phase, wrong dose and missed single dose, in that order, were the most common incidents. In prescribing, as well, wrong dose was the most common error reported, which was also the finding in earlier studies, where about one third of the errors were rated as clinically significant (Bobb et al., 2004; Keers et al., 2014). Similar to other studies, our results show that in prescribing, the second and third most common errors were missing or incomplete/inexplicit prescription, creating a risk of errors due to misunderstanding (Chang & Mark, 2009; Dickson & Flynn, 2012; Walsh et al., 2008).

### 5.2 Consequences for patients

In all, few MIs reported in the IRSs caused severe harm to patients in our study. Consequences most commonly pertained to categories B (error occurred but did not reach the patient) and C (reached the patient but no harm). A review of over 500,000 MIs in IRSs in England and Wales showed that the clinical outcome in 83.3% was no harm and in 13.3% low harm (Cousins, Gerrett, & Warner, 2012). In administration‐phase errors, most incidents pertained to category C and in prescribing‐phase errors to category B. This indicates that mistakes made by nurses more often reach the patients but also that RNs play an important role in detecting errors. Earlier studies have shown that nurses engaged in error inception practices as often as 18 times per 1,000 patient days. Critical care nurses recovered on average as many as two errors per eight‐hour shift and as few as one error per week. Perioperative nurses recovered on average 11 errors per surgical procedure (Yang et al., 2012).

When the RN does not recover the error, it can lead to severe damage to the patient (Dykes, Rothschild, & Hurley, 2010; Gaffney, Hatcher, & Milligan, 2016). Since earlier studies have proven that a considerable part of nursing injuries is ascribed to medication errors (Cronenwett et al., 2007; Makary & Daniel, 2016; Soop et al., 2009), this indicates that far from all moderate to severe MIs are reported in the IRS (Levinson, 2012; Westbrook et al., 2015).

### 5.3 Reporters’ perceptions of causes of medication incidents

Just as in earlier studies, our study found that nurses report mot incidents, not only incidents caused by themselves but also caused by other professionals (Hashemi, Khaliq, & Blakeley, 2010; Jylhä et al., 2016; Rowin et al., 2008; Schuerer et al., 2006). This is likely to indicate an underreporting of MIs by other healthcare professionals. Further, previous research has shown that severe MIs often are reported, but near misses more seldom are (Hamilton et al., 2018; Rutledge, Retrosi, & Ostrowski, 2018) and with this, missed opportunities to further investigate and take proper measures in error prevention.

Our study showed that errors in prescribing most often are reported by the person detecting the error, not the one causing it, meaning the nurses report the physicians’ mistakes. This is in line with the findings in earlier research (Jylhä et al., 2016). In effect, RNs are estimating why physicians made the mistakes (lack of knowledge), leading to difficulties in determining the actual causes of MIs in the prescribing phase. A recent study pointed out that the unequal status/position of the individual who made the error and the person reporting it can be a barrier for reporting. However, that study did not clarify whether it was nurses reporting mistakes made by another nurse or by a physician or another healthcare professional (Levine, Carmody, & Silk, 2019). Further, Jylhä et al. (2016) also found that physicians report more incidents in treatment processes and fewer incidents in medication management than other professional groups.

The reporters’ perceptions of causes show that task‐related factors were the most common, with inadequate verifying and inexplicit protocol being the most frequent. In several of the IRs, the reporter ascribed the cause to lack in knowledge, skills and abilities as well as factors in the work environment. A systematic review by Keers and colleagues describing causes of errors in administration of medication showed knowledge‐based mistakes and deliberate violations to be common causes. Errors in administration, including inadequate written communication such as prescriptions, documentation and transcription, were also described, along with perceived high workload, staff health status (fatigue, stress) and interruptions/distractions during drug administration (Keers, Williams, Cooke, & Ashcroft, 2013). A text‐mining analysis of IR data showed almost the same result, where the most effective trigger terms for identifying inadequate administration were short staffing, workload and extremely busy situation (Härkänen et al., 2020). If the work situation is stressed, perhaps omitting the step of verifying or checking for the latest protocol is a way to handle stress. Similar findings have been described concerning prescribing errors (Tully et al., 2009). Insufficient communication between colleagues and co‐workers was also an ascribed cause in our study. At many wards, a tool for structured handing over between nurses is used. Earlier studies have indicated that such a tool could improve communication (Beckett & Kipnis, 2009; Marshall, Harrison, & Flanagan, 2009).

### 5.4 Limitations

This study has its limitations. First of all, it is known that only a fraction of all incidents get recorded in the IRS (Evans et al., 2006; McBride‐Henry & Foureur, 2006; Wakefield, Uden‐Holman, & Wakefield, 2005). Thus, the material in this study represents only a limited part of medication incidents. On the other hand, the sample was large, including 19 hospitals in five of 20 CCs, which amounts to 25% of all CCs in Sweden. The consistency in the results of the study and of earlier studies might indicate that the results are applicable at a national level. Only 128 of the 775 IRs held enough information to draw conclusions about the cause of the incidents and the professional category reporting. It is possible that other causes could have been discovered if all the MI reports had been analysed, but this was not conceivable in the scope of this study. Another limitation is that the MI reports were read and categorized by the first author (MC) alone; however, the progress of each step in the analysis was scrutinized and discussed and iteratively revised between all three authors until final agreement was established. This was done to ensure credibility of the whole data analysis process (Graneheim & Lundman, 2004).

## 6 CONCLUSIONS

This study contributes to knowledge about MIs and the reporting of MIs. Getting the right medication to the right patient is a joint responsibility of RNs and physicians, and we need to look at MIs as a common problem that calls for common solutions. Further investigation for educational efforts to improve reporting, monitoring and follow‐up of MIs is warranted. The IRSs are complex systems and sometimes complicated for the users to understand and use. However, it is important not to forget the aspect of individual responsibility in medication handling to avoid the occurrence of MIs.

## CONFLICT OF INTEREST

The authors declare no conflict of interest.

## AUTHORS CONTRIBUTION

All authors contributed to the study design and manuscript writing. MC contributed to data collection and analysis. IW and MHN contributed to the study supervision and data management. All authors approved the final version for submission.

## Supporting information

Appendix S1
